# Functional and Metabolic Imaging in Heart Failure with Preserved Ejection Fraction: Promises, Challenges, and Clinical Utility

**DOI:** 10.1007/s10557-022-07355-7

**Published:** 2022-07-26

**Authors:** Matthew K Burrage, Andrew J Lewis, Jack J J. Miller

**Affiliations:** 1 Oxford Centre for Clinical Cardiovascular Magnetic Resonance Research (OCMR); Radcliffe Department of Medicine, University of Oxford, John Radcliffe Hospital, Oxford, UK; 2 Department of Physiology, Anatomy and Genetics, University of Oxford, Parks Road, Oxford, UK; 3 The PET Research Centre and The MR Research Centre, Aarhus University, Aarhus, Denmark; 4 Department of Physics, Clarendon Laboratory, University of Oxford, Parks Road, Oxford, UK

**Keywords:** Metabolic imaging, HFpEF, Cardiac metabolism, C13, PET, Heart failure, Diastolic dysfunction, Systematic review

## Abstract

Heart failure with preserved ejection fraction (HFpEF) is recognised as an increasingly prevalent, morbid and burdensome condition with a poor outlook. Recent advances in both the understanding of HFpEF and the technological ability to image cardiac function and metabolism in humans have simultaneously shone a light on the molecular basis of this complex condition of diastolic dysfunction, and the inflammatory and metabolic changes that are associated with it, typically in the context of a complex patient. This review both makes the case for an integrated assessment of the condition, and highlights that metabolic alteration may be a measurable outcome for novel targeted forms of medical therapy. It furthermore highlights how recent technological advancements and advanced medical imaging techniques have enabled the characterisation of the metabolism and function of HFpEF within patients, at rest and during exercise.

## Introduction

### The Demographics of Heart Failure

Half of patients clinically presenting with heart failure (HF) have a preserved left ventricular ejection fraction (HFpEF). Despite this, the symptoms of HFpEF are typically sufficient to cause a substantial reduction in their quality of life. Their disease follows a substantially similar course to patients suffering heart failure with a reduced ejection fraction (HFrEF), with a similar morbidity and mortality burden. HFpEF presents a large and currently unmet need for effective medical therapy and is increasingly recognised as a major and growing pathology with a complex aetiology, increasing at a rate of approximately 1% per year [1]. As its major risk factors, including hypertension, obesity, and type 2 diabetes mellitus (T2DM), are increasing in most populations, HFpEF is expected to emerge as the most prevalent HF phenotype worldwide [2]. It is worth highlighting that patients with HFpEF are as functionally limited as their counterparts with HFrEF: requiring frequent hospitalisation and living with a poor quality of life [3–5], and their overall mortality is similar to that of most HFrEF populations, with observational studies reporting a dismal 5-year survival of only 35–40% post-hospitalisation for HF—comparable to many cancers.

**Fig. 1:**
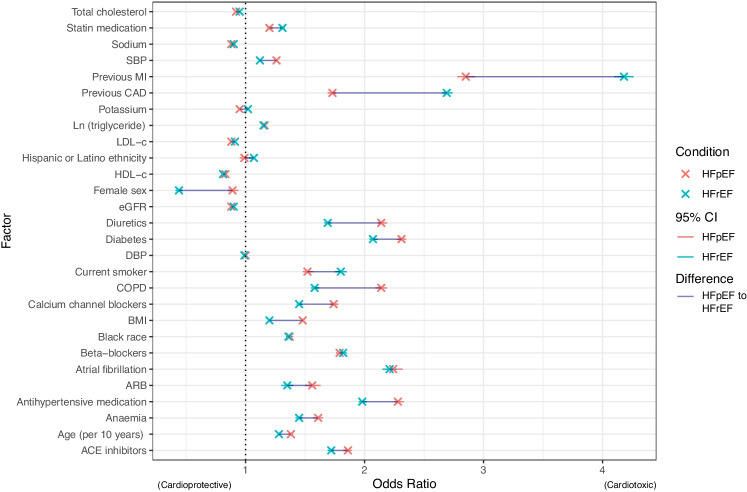
Multivariable logistic regression derived odds ratios (OR) for risk factors in developing HFpEF and HFrEF in the 1.8 million US Veterans Affairs population (comprising $n=66\,831$ HFpEF and $n=92\,233$ HFrEF patients). Data are reproduced (with permission) from Gaziano et al. [6], and have been corrected for age, sex, race, and ethnicity. A thin blue line highlights the differences between HFpEF and HFrEF. Abbreviations: ACE, angiotensin converting enzyme; ARB, angiotensin receptor blocker; BMI, body mass index (coefficient per ${5}\,{\text{kg}/\text{m}^2}$); CAD, coronary artery disease; COPD, chronic obstructive pulmonary disease; DBP, diastolic blood pressure (per 10 mmHg); eGFR, estimated glomerular filtration rate (per ${15}\,{\text{ml}/\text{min}/1.73 \text{m}^2}$; HDL-c, high density lipoprotein cholesterol (per 15 mg/dl); HFpEF, heart failure with preserved ejection fraction; HFrEF, heart failure with reduced ejection fraction; LDL-c, low density lipoprotein cholesterol (per 35 mg/dl); MI, myocardial infarction; SBP, systolic blood pressure (per 20 mmHg)

The demographic of the HFpEF patient is varied and typically comorbid. Population-based studies (e.g. the US Veterans Affairs cohort of 1.8 million individuals [6]) have reported that HFpEF patients are predominantly older women [1], with established cardiovascular risk factors such as obesity, hypertension, chronic kidney disease, coronary artery disease (CAD), anaemia, hyperlipidaemia, T2DM, and atrial fibrillation [7–9] shown via multivariable logistic regression to be associated with increased odds ratios (OR) of developing HFpEF. Whilst the existence of these risk factors is arguably unsurprising, their relative magnitudes differ at times substantially between the conditions: for example, the comparative risk for developing HFpEF following previous MI is approximately half that compared with HFrEF (OR / 95% confidence interval: 2.85 (2.78–2.93) for HFpEF compared to 4.18 (4.10–4.26) for HFrEF); with BMI and diabetes further independently hinting at a distinct mechanism of pathology (c.f. Fig. 1) on a population level alone [10].

### The Link Between Pressure, Function, and Metabolism

This information, replicated through many different patient studies, provides a strong but subtle cue to examine the molecular pathogenesis of HFpEF: its risk factors such as type 2 diabetes, obesity, and hypertension, are all indepedently associated with impaired cardiac energy metabolism [11–17].

From a fundamental physical point of view, this is self-consistent: the heart as a pump does hydraulic work whilst iterating through a pressure-volume ($P-V$) loop and, from basic thermodynamics, the integral area of that loop, $\oint P\cdot \text {d}V$, necessarily has to be equal to the stroke work undertaken in a cardiac cycle. Broadly speaking, HFpEF is a disease characterised by high filling pressures and mechanically stiff ventricles, with patients falling on a spectrum of diastolic dysfunction [18]. Approximately a third of the patients presenting with HFpEF have a normal collagen volume fraction as ascertained through endomyocardial left-ventricular biopsies [19] (the remainder raised), but all possess LVEDP, LV end-systolic wall stress, and LV stiffness modulus consistent with patients presenting with a raised collagen volume fraction. This suggests that in addition to collagen deposition, intrinsic cardiomyocyte stiffness also contributes directly to diastolic LV dysfunction in HFpEF, a finding experimentally borne out in patient groups where further biopsies are available [19, 20]. The biomechanical effect of these alterations in myocardial stiffness is a marked increase in pulmonary arterial pressure: an increase in myocardial stiffness results in both impaired LV relaxation and filling. This increases the left atrial pressure, exposing the whole of the pulmonary vascular system and ultimately the right heart to an increased pressure workload accordingly, independent of other (comorbid) associated changes in the peripheral vascular resistance that may be present in the patient. This leads to increased pulmonary transcapillary hydrostatic pressure, which drives fluid transudation, potentially increasing capillary diameter from the Young-Laplace relation, and hence leading to pulmonary congestion and breathlessness [21]. What is perhaps often under-appreciated is the role of the right heart in this process. Reduced right ventricular contractile reserve and reduced coupling of the right ventricle to the pulmonary circulation or RV-PA coupling results in increased right-sided pressures, with right atrial dilatation and increased right atrial and systemic venous pressures. This exacerbates pulmonary congestion by reducing clearance of lung water via pulmonary lymphatics [22–25].

Together with an increase in myocyte and ventricular stiffness (i.e. mechanically increasing stroke work), the metabolome in HFpEF patients is significantly altered, and again differentially altered in comparison to HFrEF patients. Plasma metabolomics reveals that, accounting for differences in blood biochemistry known to be due to comorbidities such as T2DM, HFpEF is associated with indices of increased inflammation and oxidative stress (such as higher levels of hydroxyproline and symmetric dimethyl arginine, alanine, cystine), impaired lipid metabolism (lower lysophophatidylcholine, potentially altered coupling between glucose oxidation and glycolysis [26]), increased collagen synthesis, and downregulated nitric oxide signalling. Together, these findings suggest a more predominant systemic microvascular endothelial dysfunction and inflammation linked to increased fibrosis in HFpEF compared with HFrEF [27].

**Fig. 2:**
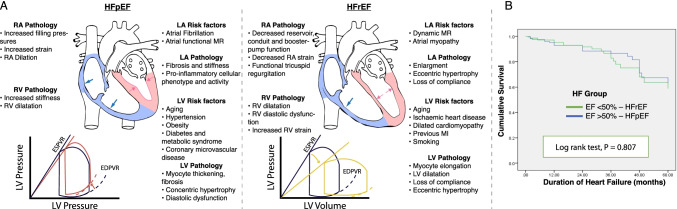
**A** Taken together, these factors are sufficient to delineate HFpEF as a very distinct disease from HFrEF. As detailed by Guazzi et al. [28], in HFpEF, concentric myocyte remodelling with increased left ventricular (LV) diastolic stiffness, atrial functional MR, and a stiff left atrium (LA) are the major driving forces to alveolar-capillary stress failure and vascular remodelling, with corresponding differential changes in the end-systolic/end-diastolic pressure-volume relation (ESPVR/EDPVR) compared to healthy controls (shown in blue). **B** It is worth further stressing that, although not typically caused by an ischaemic origin, HFpEF has a similar mortality to HFrEF following MI, as illustrated here in an “all-comers” Ethiopian cohort HF study reporting outcomes on all HF patients treated at the University of Gondar Referral Hospital between 2010 and 2015 [29]

As summarised in Fig. 2A, HFpEF therefore remains a disease that is (a) prevalent and expected to become increasingly so; (b) complex and necessarily associated with numerous comorbid pathologies that may help define its molecular milieu; (c) deeply connected with the biophysical mechanics of the heart; and (d) a disease with a high mortality and morbidity (Fig. 2B).

## Cardiac Metabolism in Heart Failure

### The Heart as a Metabolic Omnivore

The healthy heart has an enormous energy requirement and consumes more energy and oxygen than any other organ [30]. It continuously produces large amounts of adenosine triphosphate (ATP), which is necessary to sustain both active myocardial contraction and active diastolic relaxation. This is achieved by metabolising a variety of fuels (primarily fatty acids and glucose with additional contributions from lactate, ketones, and amino acids) and oxidative phosphorylation within the cardiac mitochondrial respiratory electron transport chain [31, 32]. In health, the bulk of mitochondrial ATP production, which contributes approximately $\sim 95\%$ of myocardial ATP requirements, is derived from fatty acid oxidation ($\sim 40$–$70\%$), with the remainder originating from glucose and lactate via the oxidation of pyruvate [32, 33]. Only about 5% of myocardial ATP demand is met by glycolysis, which does not require oxygen [32, 34, 35]. The heart demonstrates a degree of metabolic flexibility and is able to utilise different energy substrates under different conditions to maintain function, although this may become lost in the failing heart [36]. Given the reliance of actin-myosin interactions on ATP and oxygen availability, it is clear that disruption in cardiac energy metabolism pathways and altered myocardial energetics have significant implications for cardiac function and the pathogenesis of heart failure [30, 32].

### The Failing Heart and Its Fuels

As outlined above, heart failure presents a major societal impact, is increasingly prevalent, and is associated with substantial morbidity and mortality [37]. However, the role of cardiac energy metabolism is perhaps more intuitive in the pathophysiology of HFrEF, in that it is easier to conceptualise that the failing heart with contractile dysfunction is starved of fuel [30]. Impaired cardiac energy metabolism as demonstrated with non-invasive phosphorus magnetic resonance spectroscopy (${}^{31}$P-MRS) has been shown to correlate with the degree of heart failure and predict mortality in patients with HFrEF due to ischaemic and dilated cardiomyopathy [38–41]. In contrast, HFpEF remains challenging to diagnose, vastly heterogeneous in terms of pathophysiology and clinical risk factors and, until recently [42], had few therapeutic options [43]. Because of this, there is increasing interest to more carefully phenotype individual patients with HFpEF [44]. Recent basic scientific and translational studies have provided further evidence that impaired cardiac energy metabolism also plays a central role in the pathogenesis of HFpEF, with alterations in lipid metabolism, and an increase in mitochondrial stress and ROS together with other inflammatory-related changes in mitochondrial function [45–49]. This is plausible, given that active diastolic relaxation has the greatest energy demand of any phase of the cardiac cycle [50]: rather than a metabolic disorder of contraction, HFpEF is, if anything, a disease of relaxation (but one that is distinct from diastolic dysfunction alone [21]) and moreover one with few universally recognised animal models of the condition, in contrast to HFrEF in which ischaemia-reperfusion models of MI progress routinely to HFrEF. In both conditions, cardiac dysfunction further manifests as a series of profound shifts in the utilisation of fuels through specific pathways, as summarised in Table 1, which potentially appear to be distinct between HFpEF and HFrEF.

**Table 1:** A brief summary of some of the main metabolic changes associated with HFpEF (in comparison with HFrEF). The detailed metabolic changes that occur in heart failure are complex, and are dependent not only on the severity and type of heart failure present, but also on the coexistence of common comorbidities such as obesity and type 2 diabetes, and reviewed in detail elsewhere [70]

Metabolic pathway	HFrEF	HFpEF
Fatty acid oxidation	Increased in humans, measured in vivo [51, 52]	Reduced in humans *in vivo* [51, 53, 54] and in rodents [55] measured via *ex vivo* Langendorff perfusion
Glucose oxidation	Reduced in mice, measured via *ex vivo* perfusion [56, 57]	Reduced in LV biopsy [58] from left-ventricular assist device (LVAD) patients with advanced HF and in rodents [59–61] measured via Langendorff perfusion
Glycolysis	Unchanged [62] or increased [63] in Langendorff-perfused rat heart	Increased in LVAD patients after biopsy [64]; also increased in rats [59].
Coupling between glucose oxidation and glycolysis	Unchanged in the perfused hearts of mice [56] or reduced in rats [63]	Reduced in human LVAD patients [64] and in mice [65].
Ketone body oxidation	Unknown, insufficient data	Increased in human advanced HF patients [66, 67] as determined either via biopsy at the time of LVAD implantation or transplantation
Branched chain amino acid metabolism	Unknown, insufficient data	Impaired in humans [68] and mice [68, 69] as measured via biopsy

Multiple cardiometabolic and other risk factors for HFpEF, such as T2DM, obesity, and hypertension, are all independently associated with impaired cardiac energy metabolism, and can be shown through magnetic resonance spectroscopy studies in human patients and volunteers under a variety of cardiometabolic states to quantitatively affect cardiac energetics [11–17]. Diastolic dysfunction is also associated with impaired myocardial energetics in translational and clinical models, but represents a more widely spread disease in patients [71, 72].

It is therefore clear that metabolic modulation may thus be a promising therapeutic strategy for the treatment of heart failure in appropriately phenotyped patients [32, 73]. As detailed subsequently, novel metabolic and functional imaging techniques may help identify those patients who could derive most benefit from such strategies, as well as act as appropriate tools to use in future clinical trials.

## Inflammation and HFpEF

Alongside impaired myocardial energetics, there is a strong body of pre-clinical and clinical evidence that HFpEF is a multi-system disease characterised by systemic inflammation that affects the myocardium, skeletal muscles, vasculature, and kidneys [74], ultimately resulting in increased left ventricular myocardial stiffness and fibrosis. This is one of the hallmarks of HFpEF physiology, where increased myocardial stiffness and impaired diastolic relaxation results in high LV filling pressures and exertional breathlessness. It has been proposed that systemic inflammation is linked to comorbidities associated with HFpEF, particularly diabetes mellitus and obesity, which are associated with a systemic proinflammatory state, haematopoietic activation and systemic leukocytosis [75], and the subsequent development of myocardial stiffness and fibrosis [76].

### Titin as a Link Between Inflammation and Stiffness

Paulus et al. have presented a clear series of pathophysiological mechanisms that link systemic inflammation and myocardial stiffness [74]. Metabolic load associated with comorbidities, such as obesity, diabetes, and chronic kidney disease, induces proinflammatory signalling and systemic inflammation as evidenced by raised plasma levels of inflammatory cytokines [77–80]. Haemodynamic load, as seen in arterial hypertension, also activates proinflammatory and profibrotic signalling pathways [74]. Increased haemodynamic load is sensed by cardiomyocytes, fibroblasts, and resident macrophages resulting in a cascade of structural remodelling and leukocyte recruitment that promotes fibroblast activation and collagen deposition, thereby increasing myocardial stiffness [74]. Modifications in the titin protein are a major contributor to cardiomyocyte stiffness. Titin is the largest protein in the body, spans multiple regions within the human sarcomere, and is responsible for diastolic distensibility. It consists of two main isoforms, the short stiff N2B isoform and the longer, more compliant N2BA isoform. Post-translational titin oxidation or phosphorylation modulates distensibility and contribute to the high myocardial stiffness seen in HFpEF. The N2BA/N2B titin isoform ratio is lower and the N2B isoform is hypophosphorylated in HFpEF [81, 82].

**Fig. 3:**
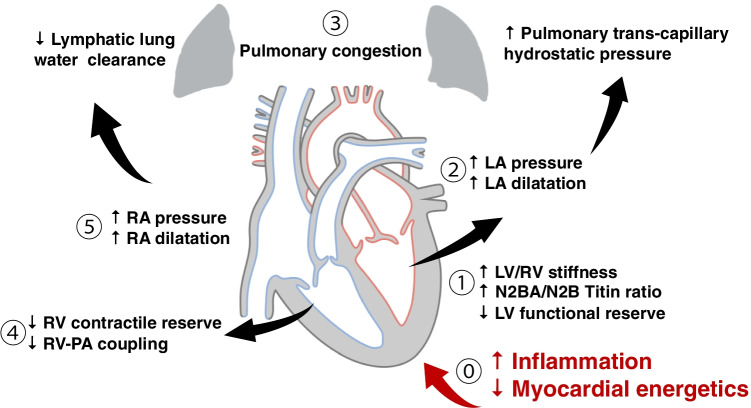
A very brief schematic overview of the currently understood pathogenesis of HFpEF. It is important to remember that the HFpEF syndrome is a biventricular process and that the contributions of the right heart are equally important. (1) Increased left ventricular stiffness, and a decreased functional reserve, results in (2) increased left-sided filling pressures with left atrial dilatation and increased left atrial pressure. This leads to increased pulmonary transcapillary hydrostatic pressure, which drives fluid transudation, potentially increasing capillary diameter from the Young-Laplace relation, and resulting in pulmonary congestion (3). Similar maladaptive right heart processes may also occur in parallel. (4) Reduced right ventricular contractile reserve, and reduced coupling of the right ventricle to the pulmonary circulation or RV-PA coupling, results in increased right-sided pressures, with right atrial dilatation and increased right atrial and systemic venous pressures (5). This exacerbates pulmonary congestion by reducing clearance of lung water via pulmonary lymphatics. The underlying pathogenesis of HFpEF is still the subject of ongoing research, but pro-inflammatory changes result in a mechanical increase in the stiffness of the myocardium mediated through alterations in collagen deposition and the biophysics of titin, which occurs concomitantly with metabolic changes leading to an increased oxidative stress on the myocyte

Additionally, reactive oxygen species (ROS) cause formation of disulfide bonds within titin, also contributing to increased cardiomyocyte stiffness [83, 84]. Changes in myocardial collagen homeostasis also lead to abnormalities of the extracellular matrix, resulting in myocardial fibrosis and stiffness [76]. This has been evidenced by an increase in collagen volume fraction on myocardial biopsy in patients with HFpEF [84, 85]. Finally, the myocardial accumulation of degraded proteins, which occurs due to inflammation-triggered expression of inducible nitric oxide synthase in cardiomyocytes, is also thought to drive myocardial stiffness [45, 76]. This increase in stiffness directly causes haemodynamic effects that lead ultimately to the development of the symptoms of the disease, as summarised in Fig. 3.

**Table 2:** Human biopsy studies in HFpEF reveal a complex, multifacted disease with increased collagen deposition, alterations in the ECM, and a significant inflammatory component. Reported n is for HFpEF patients in each study; studies listed have substantially different designs and control arms. Abbreviations: ECM, extracellular matrix; EM, electron microscopy; FA, force analysis; HM, histomorphometry; IF, immunofluorescence; IHC, immunohistochemistry; RT-PCR, real-time reverse transcription-polymerase chain reaction

Patient population	*n*	Assays	Findings	Ref.
“All comers” advanced HFpEF	108	Histology	Fibrosis in 93% of patients; hypertrophy in 88%; inflammation $1.9\times$ higher compared to controls (as measured by CD68 expression); amyloidosis in 14%	[86]
CABG	16	Transcriptomics	743 differentially expressed genes; alterations in ox phos, extracellular matrix proteins, titin, potassium voltage-gated channels	[87]
Suspected cardiomyopathy, HFrEF, and HFpEF	36	Western blotting, IHC, IF	Increased E-selectin and intercellular adhesion molecule-1 expression (HFpEF compared to HFrEF); increased NADPH oxidase 2 expression in macrophages and endothelial cells	[88]
Suspected cardiomyopathy	27	FA, HM, isoform and phosphorylation assays	Stiffer myocardium, N2BA / N2B titin isoform ratio reduced, and N2B isoform hypophosphorylated in HFpEF	[81]
Worsening HF, suspected cardiomyopathy	22	FA, HM and EM	Increased collagen content, higher cardiomyocyte diameter, higher passive force, stiffer	[82]
Restrictive cardiomyopathy	12	FA, collagen volume fraction	Stiffer myocytes, often higher collagen volume fraction	[19]
NYAHA class II+	20	RT-PCR, collagen assay, histology, TGF-$\beta$ ELISA	Increased collagen content, decreased matrix metalloproteinase-1 (which is the primary collagenase of the heart and removes ECM), TGF-$\beta$ positive inflammatory cells present	[89]
Hypertensive CABG patients	22	FA, collagen assay, isoform and phosphorylation assays	Increased passive myocardial stiffness; collagen-dependent and titin-dependent stiffness	[84]

It is worth reflecting that many studies which have provided mechanistic and molecular detail on the mechanobiology of HFpEF have done so through the acquisition of myocardial biopsy samples taken from patients listed for operations related either to their condition or their comorbidities, typically at the more severe end of the phenotype. These samples provide histological evidence supporting the twin hypotheses of both differential extracellular matrix content (with HFpEF patients having higher titin expression in their myocytes and a higher collagen content in the surrounding extracellular matrix), leading to mechanically stiffer myocytes with a stronger passive force per unit area ($F_\text {passive}$); and inflammation associated with HFpEF. In addition, endomyocardial biopsy provides a gold-standard method to diagnose or rule out cardiac amyloidosis, a prevalent aetiology of “HFpEF” that is mechanistically distinct, caused by the extracellular deposition of autologous protein as amyloid fibrils from a variety of more than 30 molecular sources [90] that initially presents as a restrictive cardiomyopathy characterized by progressive diastolic dysfunction that subsequently becomes systolic biventricular dysfunction with oft-fatal arrhythmia. A summary of histological findings in HFpEF is shown in Table 2, which are consistent with the pattern described above. Whilst this tissue is highly valuable, and can be subject to a large number of related differential analyses, it is prudent to be mindful of the fact that human biopsy samples are typically only obtained from patients at the more developed end of the disease spectrum and for whom the surgical intervention is otherwise justified. This selection bias combined with the risks and cost of biopsy motivates the investigation of both non-invasive, or less-invasive diagnostic techniques such as imaging methods, and furthermore the scientific study of less severe disease.

### Imaging Inflammation

The ability to non-invasively characterise myocardial inflammation and fibrosis has important implications for management. Emerging imaging tracers using hyperpolarized magnetic resonance or positron emission tomography enable non-invasive assessment of major cardiac leukocyte populations, including macrophages [91, 92]. Novel PET tracers targeting the cardiac stroma including collagen could be combined with cardiovascular MR for a complete assessment of the extracellular volume fraction. A recent study which identified patients with HFpEF and myocardial fibrosis on cardiovascular magnetic resonance imaging showed that the oral antifibrotic agent pirfenidone may reduce myocardial fibrosis [93]. The effects of this on clinical outcomes and patient symptoms still needs to be confirmed. Other immune-mediated conditions may more directly cause cardiac inflammation and heart failure symptoms. Multiple systemic autoimmune or immune-mediated disorders may cause myocarditis, where inflammation is the primary driver of cardiac dysfunction, while this is most commonly attributable to viral infection [94]. Identification of active primary cardiac inflammation is important as patients are far more likely to respond to immunosuppressive therapies. Advanced myocardial tissue characterisation techniques have shown considerable promise in the detection of myocardial inflammation [95], having identified cardiac involvement in a wide variety of systemic conditions [96–104].

## Functional Imaging and HFpEF

It is therefore highly desirable to attempt to simultaneously ascertain both the function of the heart and measures of its metabolome and microstructure in the context of patients who may well be suffering from relevant comorbid diseases (e.g. T2DM). It is therefore recognised that, in a clinical context, the diagnosis of HFpEF remains challenging, with several diagnostic criteria proposed by learned bodies and in clinical trials [18].

Imaging approaches span a spectrum of complexity, invasiveness, availability, and cost, and these competing concerns have to be balanced against either the scientific rationale for their engagement, or the clarity which they could add to the diagnosis of a condition that exhibits a high misdiagnosis rate [105, 106]. Furthermore, HFpEF is a condition where comparatively subtle physiological changes can be manifest even at the early stages of the disease [18]. The haemodynamic response of the heart to increased myocardial stiffness includes left atrial dilatation (with a volume index $>{32}\,\text {ml/m}^2$ shown to increase the risk of cardiovascular mortality), and a reduction of diastolic function as typically quantified by the mitral E velocity ($>{90}\,\text {cm/s}$) and septal e’ velocity ($<{9}\,\text {cm/s}$) on echocardiography, or equivalently an increase in their ratio ($>9$). Whilst some degree of left ventricular hypertrophy is supportive of HFpEF, its absence does not exclude its diagnosis [18].

What is perhaps under-appreciated is the variability present within the condition and the degree to which it can be masked by existing co-morbidities or be confused with other forms of heart failure; one study by Kanagala et al. [107] on 154 patients with a provisional HFpEF diagnosis underwent a comprehensive CMR examination (stress/rest perfusion, cine function, and late gadolinium enhancement). Forty-two patients (27%) were found to have an alternative diagnosis (coronary artery disease, microvascular dysfunction, hypertrophic cardiomyopathy, or constrictive pericarditis), with worse outcomes.

### Cardiovascular MR in HFpEF

Cardiovascular magnetic resonance (CMR) is the gold-standard imaging modality for assessing atrial and ventricular volumes and accurately quantifying ejection fraction (in contrast to echocardiography, which suffers both from an increased measurement variability and systematic bias in underestimating cardiac volumes, owing both to increased inter- and intra-observer variability and the conventional assumption that the LV forms a prolate ellipsoid when its mass is estimated through linear measurements made of the wall on the parasternal long-axis view [108–110]). As a consequence, although the full role of CMR for diastolic function assessment is evolving, it currently allows accurate assessment of the structural changes associated with HFpEF, such as left atrial (LA) enlargement, and pathological LV hypertrophy, along with mitral inflow and pulmonary venous flow velocity. These MR flow measurements are obtained via phase-contrast (PC) methods, wherein a bipolar gradient of magnetic field is applied along a given axis, and a change in the phase of the MR signal produced in proportion to the velocity of blood flowing along it. For the most part, the interpretation of these measures largely follows that of Doppler echocardiography, with a comparatively more coarse temporal resolution and a requirement to acquire data over several heartbeats as opposed to in real-time, meaning that CMR measurements of flow are hence more susceptible to arrhythmias. However, in addition to the measurement of mitral and pulmonary flow in one direction, it is possible to quantify the three-dimensional velocity vector field of blood through with CMR, using “4D-flow” sequences in which the direction of the spatial magnetic field gradient that encodes flow information in the phase of the MR signal is varied in 3D space. Over the course of a longer, more complex acquisition, whole-heart flow patterns can be obtained throughout the cardiac cycle which can both reveal complex valvular insufficiency, retained residual blood volume, and the degree of vorticity and turbulent flow in diastole [111, 112]. HFpEF patients are understood to have increased diastolic inflow vortex strength and greater turbulent flow, again consistent with a biomechanical dysfunction of pumping function [113], leading to complex and subtly different responses to fluid-dynamical alterations brought about by, for example, the effect of a presence of an interventricular septal shunt or vasodilation, compared to either healthy individuals or those with HFrEF [114, 115].

Additionally, diastolic dysfunction can also be inferred through alterations of the filling dynamics of each chamber of the heart. The increased availability of dedicated semi-automatic cardiac image analysis and segmentation software (oft using “AI” algorithms in the process) has recently enabled these to be computed relatively more rapidly by those reading the images, removing the requirement to manually segment, e.g. endo- and epicardial contours for the quantification of the LV time-volume filling curve. The resulting peak filling rates and time to peak filling can therefore easily be obtained. Both time to peak filling, peak filling rate, and other novel indices aimed at quantifying these curves have been demonstrated to be of utility in quantifying left ventricular diastolic dysfunction [116].

Furthermore, myocardial strain (and inferred stiffness) can be directly be determined through feature tracking or tagging techniques—which can either be determined post hoc after the acquisition of a conventional cine image or by the use of the spin-physics behind MR to impose structured tags within the myocardial tissue that then deform over time [117]. These thus therefore infer the motion of individual voxels of myocardial tissue, either by imprinting upon the physical myocardium or by estimating its motion in the image domain. As strain is effectively defined as extension over original length, longitudinal, radial, and circumferential strain can be estimated for the heart by the use of either a 2D or 3D mathematical model of deformation [118], once a computational model of the deformation over time of each individual voxel has been determined. This process remains an active research area for both techniques, owing to the fact that multiple complex cardiac motions may result in the same observed deformation [119, 120]. The utility of the technique in HFpEF comes from that fact that, if considered as a rigid body, a stiffer myocardium will be unable to deform as much under the same force, and increased myocardial stiffness can be quantified directly (c.f. Table 3). Perhaps reflecting this, multi-layer feature-tracking derived strain metrics have thus been shown to correlate with NT-proBNP and effectively be able to diagnose HFpEF in a signal scan with an 89% sensitivity and a specificity of 100% [121], and global longitudinal strain has itself been associated with mortality and other hard cardiovascular outcomes in HFpEF [122].

**Table 3:** Widely available cardiovascular magnetic resonance techniques permit the detailed assessment of myocardial function and some biophysical properties of the myocardium

Name	Example image	Physical basis	Indication
CINE MR		Conventional CINE imaging acquires multiple frames throughout the cardiac cycle by the use of either a gradient-echo based or SSFP-based readout. Bright blood contrast is provided predominantly by the fresh magnetisation in blood inflowing into the slice.	A wide variety of standard cardiac anatomical parameters are readily determined by CINE MR, such as whole-heart mass and morphology. Dynamic tracing of the volume-time curve permits the determination of measures of chamber filling and emptying and flow rates.
Atrial volumes		Atrial volume-time curves are determined from CINE MR images	Atrial dilatation is indicative of HFpEF, and impaired RV early filling may be compensated by increased RA booster pump function. Impaired LA conduit function is hypothesised as a distinct feature of HFpEF.
Strain		Either (a) a spatially periodic magnetisation depleting “tagging” RF pulse is played, superimposing a regular grid of grey on the heart that deform throughout the cardiac cycle (the gold standard); or (b) image-domain points on a previously acquired CINE image are tracked (“feature-tracking”). A model of strain is computationally built from the results.	Changes in radial and circumferential strain in diastole are highly indicative of a stiffer heart and alteration with otherwise normal morphology may be a marker of early disease.
T1 mapping		The nuclear spin-lattice relaxation time, $T_1$, is measured, typically by performing an inversion-recovery experiment with an inversion RF pulse followed by a series of low-flip angle Look-Locker readout sections at different times after inversion. The injection of gadolinium and reacquisition of the map permits the estimation of myocardial extracellular volume (ECV).	Changes in myocardial T_1_ are associated with cardiac amyloidosis, and (more weakly) of HFpEF in general. ECV is strongly correlated with adverse outcomes in HFpEF cohorts.
PC flow		Bipolar gradients are used to encode the velocity of flowing blood into the phase of the MR signal. Phase-contrast methods can therefore reconstruct blood flow exactly along a given axis (typically into/out of the slice plane) or, at the expense of acquisition time, in 3D (leading to “4D flow”, a time-resolved acquisition).	Mitral flow imaging is a sensitive measure of regurgitation and can easily quantify early (E) and late (A) diastolic flow, indicative of diastolic dysfunction similar to echo.

Finally, it is possible to use CMR to assess fibrosis directly through either late gadolinium-enhancement MR, or the increasingly available technique of T_1_ mapping with and without gadolinium and the concurrent estimation of the extracellular volume (ECV) fraction [116]. These related methods utilise the known fact that gadolinium contrast agents extravasate and are retained in interstitial spaces. Whilst conventionally used for the comparatively routine detection of fibrotic scar (typically following myocardial infarction) through late gadolinium-enhancement MR, by measuring the nuclear $T_1$ time of the myocardium pre and post the addition of gadolinium subtle, not visually obvious differences in $T_1$ and hence gadolinium uptake can be determined. If the patient’s haematocrit is known, their extracellular volume fraction can be correspondingly estimated, and expressed in intuitive units of per cent [123]. An increase in ECV is almost always due either to excessive collagen deposition or cardiac amyloidosis, and ECV has been shown to act as an independent predictor of intrinsic LV stiffness in HFpEF patients as measured via invasive pressure-volume loops [124]. Fortunately for the differential diagnosis of cardiac amyloidosis, the appearance of global, subendocardial late gadolinium is characteristic [125], as is an inability to null the enhanced signal in an inversion-recovery late gadolinium MR scan, and an increase in the native $T_1$ of the amyloid myocardium [90, 126]. These CMR changes appear to be present for both AL and ATTR forms of cardiac amyloidosis, highlighting that CMR is a complementary technique to SPECT imaging with the bone-seeking diphosphate radionuclide $^{99m}$Tc-DPD, a sensitive scintigraphy tracer that is highly effective at detecting the ATTR form of cardiac amyloidosis at an early stage, but only effective in approximately a third of patients with cardiac AL amyloid [90].

Advanced forms of proton cardiac imaging, therefore, have an important and growing role in the diagnosis of HFpEF from a clinical perspective, able to probe and quantify both the morphological adaptations in the HFpEF heart arising from increased filling pressures and impaired diastolic relaxation, and also probe changes due to increased mechanical stiffness and altered collagen content. Furthermore, compared to the use of echocardiography alone, the wider availability of CMR may help address the underdiagnosis of cardiac amyloidosis as a prevalent aetiology of “HFpEF” given the practical limits of the accessibility of complementary imaging (i.e. $^{99m}$Tc-DPD SPECT-CT) for amyloid specifically.

### Exercise (In-)Tolerance

**Fig. 4:**
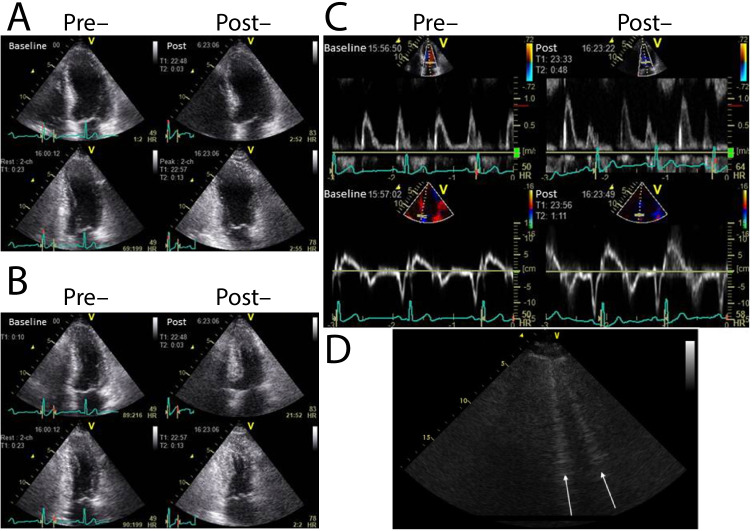
Echocardiography prior to and post exercise can reveal comparatively subtle increases in diastolic dysfunction. Reproduced here is the case of a 75-year-old male with current exertional dyspnoea and a history of percutaneous coronary interventional therapy. Wall motion analysis revealed no exercise-induced wall motion abnormality in either view (**A**, **B**). The measured mitral flow pattern and tissue Doppler was consistent with that of delayed relaxation (**C**) and E/E’ increased from 14 at rest to 16 with exercise [127]. Similarly, hyperechoic b-lines that arise in the lung from transudated fluid (**D**) that can be detected in HFpEF patients with dyspnoea, with good prognostic power [128]

**Fig. 5:**
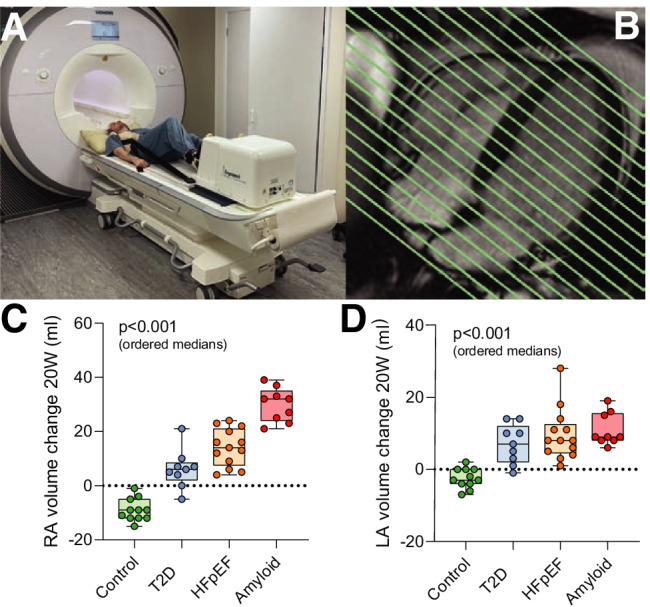
In an “in-magnet” CMR exercise study using a stepping ergometer (**A**) with whole-heart coverage (**B**) during 20 W submaximal exercise on a population of patients spanning the spectrum of diastolic dysfunction, it was found that the absolute change in right (**C**) and left (**D**) atrial volumes during exercise was highly maladaptive in HFpEF: greater dilation during exercise is suggestive of increased filling pressures [137]

Together with functional changes at rest, HFpEF, like other forms of heart failure, is associated with a significant decrease in the exercise reserve of the patient. This arises as a biophysical consequence of increased LV stiffness: during hyperaemic stress there is an inability to augment the left-ventricular end-diastolic volume despite increasing the left-ventricular end-diastolic pressure for a given cardiac workload. The outcome of this is an increased stress on both the pulmonary vasculature and the heart as a whole: as is well known in HFrEF, exercise acts therefore as a cardiopulmonary stress test under which pathology may reveal itself in HFpEF as well, with patients with early-stage HFpEF displaying symptoms and invasively measured filling pressure increases only present on exertion [129, 130]. Exercise testing with concurrent echocardiography (ExE) has been used to provide further useful pieces of information that are of relevance in the diagnosis of HFpEF. Firstly, the awareness and perception of dyspnoea may be highly variable between patients and value therefore added by an objective measure both of cardiac parameters and exercise capacity [131–133]. Secondly, it is possible to acquire myocardial E/e’ during exercise, which has been shown to increase under stress through a study of $n=436$ patients presenting with fatigue or dsypnoea and considered for the diagnosis of HFpEF [129] (as illustrated in Fig. 4A-C). Thirdly, the formation of hyperechoic “B”-line artefacts is directly detectable in the lungs of patients with acute pulmonary congestion [134], and shown to correlate with B-type natriuretic peptide (BNP), respiratory rate, clinical congestion, and systolic pulmonary arterial pressure (Fig. 4D) as a measure of pulmonary congestion. In HFpEF, the development of pulmonary congestion upon exercise is mostly concomitant with exercise-induced worsening of diastolic function [128], and CMR measures of cardiac function during exercise indicate that the HFpEF patient suffers atrial dilatation, indicative of higher filling pressures (Fig. 5). Tricuspid regurgitation has additionally been shown to be present on ExE, which, together with E/E’, can predict future mortality [135, 136].

## Metabolic Imaging Methods

These profound changes in the morphology, function, and structure of the heart in HFpEF occur concomitantly with alterations in metabolism and a shift in substrates used by the heart, as inferred by biopsy and metabolomic approaches outlined previously in Table 1. These invasive techniques necessarily are difficult to apply to a larger number of patients and are typically reserved either to studying preclinical models or patients who have effectively reached the end stage of the disease and undergo surgery either for LVAD implantation or transplant. In contrast, non-invasive or less-invasive molecular imaging techniques have recently been applied to both confirm or discover that these changes exist *in vivo* and at an earlier stage of the disease. All of these techniques provide a consistent picture of the disease becoming unmasked under exercise stress, summarised in Table 4.

**Table 4:** A summary of reported cardiovascular abnormalities in exercising HFpEF patients

Modality	Measurement found in HFpEF	Relevance	Refs.
Echocardiography	Mitral E/E’ > 15; increased right ventricular systolic pressure; tricuspid regurgitation velocity >3.4m/s	ExE measures correlate with invasively measured pulmonary capillary wedge pressure (PCWP) and form part of the ESC diagnostic criteria (HFA-PEFF score)	[18, 138]
	Mitral regurgitation; $\downarrow$ left atrial strain augmentation	Indicative of right ventricular dysfunction and inefficient ventilation during exercise	[139]
Cardiac magnetic resonance imaging	$\downarrow$ Diastolic filling rate; $\uparrow$ left and right atrial volumes; $\downarrow$ right ventricular ejection fraction augmentation during exercise; $\uparrow$ lung proton density (water); $\downarrow$ left atrial ejection fraction; $\downarrow$ left atrial long axis strain; alterations in T_1_ and ECV. Decreased PCr/ATP ratio.	Cardiac functional patterns linked to degree of impaired resting energetics (measured by the ${}^{31}$P PCr to ATP ratio)	[116, 137]
Invasive haemodynamic measurements	PCWP $\ge$25.5 mmHg/W/kg; PCWP cardiac output slope >2 mmHg/l/m; supine PCWP $\ge$ 25 mmHg	Abnormally high exercise PCWP are confirmatory/diagnostic of HFpEF and have been associated with adverse cardiovascular outcomes	[140, 141]
Radionuclide ventriculography	$\uparrow$ Time to peak filling; $\downarrow$ $\Delta$ left ventricular ejection fraction augmentation; $\uparrow$ arterial to left ventricular end systolic elastance (vasculoventricular coupling)	In HFpEF during exercise, the active relaxation phase of diastole lengthens; shortens in controls	[72]
ECG	Chronotropic competence	Abnormal heart rate augmentation relates to degree of exercise intolerance	[142]
Cardiopulmonary exercise testing	$\downarrow$ Peak VO$_2$; $\uparrow$ VO$_2$ recovery kinetics; $\uparrow$ VE/VCO$_2$ slope	Measures have been shown to predict cardiovascular outcomes among patients with HFpEF	[143, 144]

*Abbreviations: HFA-PEFF*, Heart Failure Association-PEFF with “P” standing for pre-test assessment, “E” echocardiography, “F” functional testing, and “F” final aetiology; *PCWP*, pulmonary capillary wedge pressure; *VE/VCO*$_2$, ratio of minute ventilation to carbon dioxide elimination; *VO*$_2$, oxygen uptake [145﻿]

Broadly speaking, CMR with nuclei other than protons, X-nuclei MR, and radionuclide imaging techniques are those that are able to best interrogate these changes in humans. Phosphorus-31 cardiac magnetic resonance spectroscopy is an established X-nuclear MR technique that is able to quantify the presence and concentration of ${}^{31}$P-containing moeities within the human heart, most notably phosphocreatine (PCr) and ATP. This reflects the energetic status of the heart as phosphocreatine acts as a labile energy buffer and the endpoint of multiple metabolic pathways. Accordingly, the PCr/ATP ratio is well known to be lowered in advanced heart failure [30], diabetes [146, 147], and HFpEF [148].

**Fig. 6:**
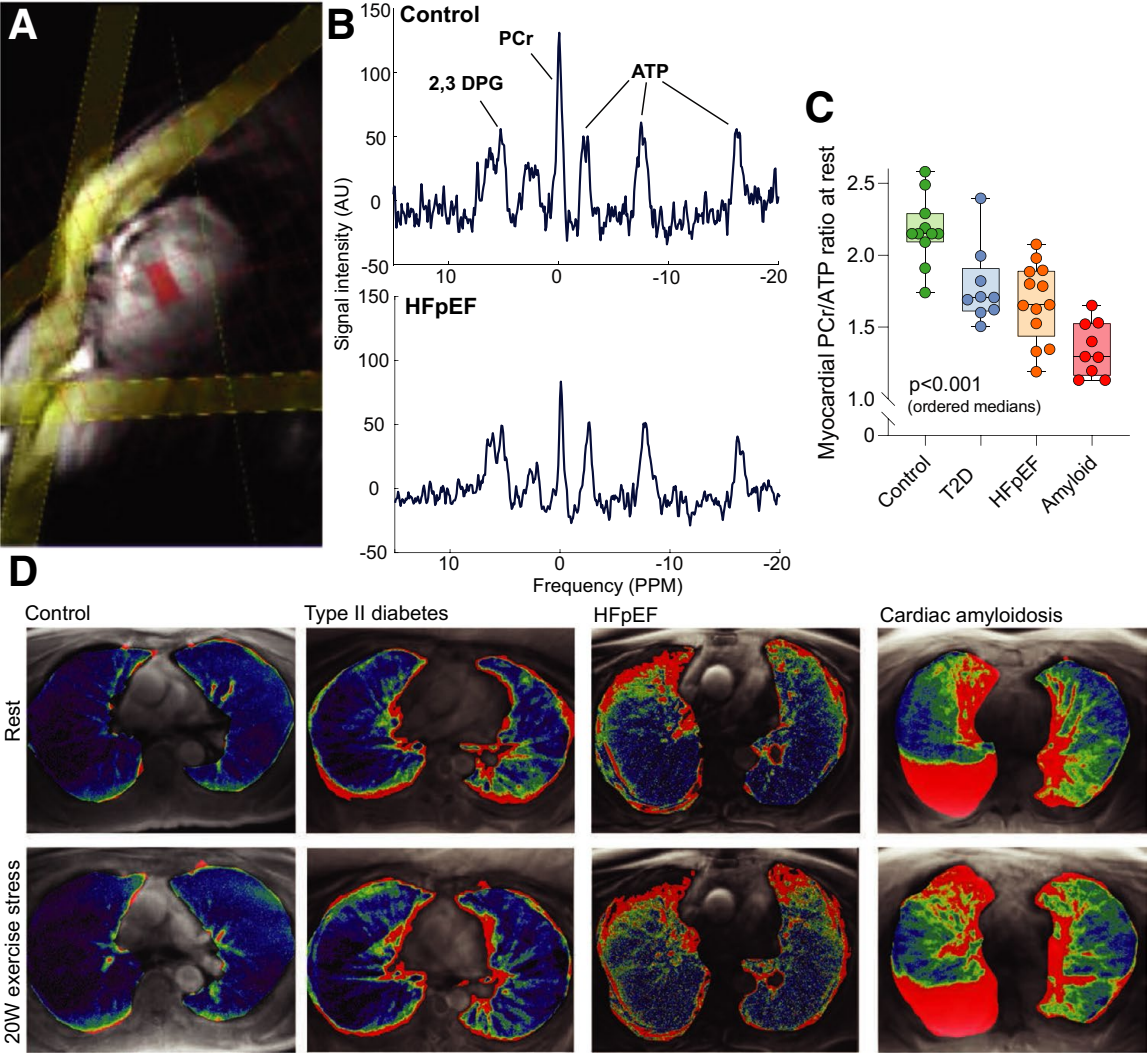
Patients with increasing diastolic dysfunction progressively dilated atrial volumes, most likely as a result of increased filling pressures as alluded to in Fig. 3. ${}^{31}$P-magnetic resonance spectroscopy (obtaining from the mid interventricular septum**A** spectra showing the presence of high-energy metabolites such as phosphocreatine (PCr), three resonances corresponding to the three ${}^{31}$P nuclei in ATP; and 2,3-diphosphoglycerate, **B**). This subsequently revealed in that these patients a discrete energetic deficit (i.e. a reduction in the detectable PCr/ATP) that was progressively worse across the groups (**C**). This was accompanied by an increase in detected pulmonary fluid as detected by a novel proton-density mapping MR sequence (**D**, [137])

Interestingly, it is possible to perform exercise experiments within the confines of the bore of the MR scanner, permitting the determination of PCr/ATP at rest and during submaximal exercise, together with the observation of functional changes in cardiac haemodynamics [148]. This therefore highlights multinuclear CMR as a powerful technique with the ability to simultaneously interrogate the energetic status of the heart through ${}^{31}$P-spectroscopy, and probe functional and microstructural methods as outlined above in the same scan session. Some authors have developed magnet-safe exercise devices, together with advanced cardiac and pulmonary imaging CMR sequences (retrospectively gated compressed-sensing cine; and a novel radial ultrashort echo time lung water proton density mapping sequence) that permit the quantification of cardiac function and pulmonary free fluid during exercise ([137]; summarised in Fig. 6). It was found that under a constant low workload of 20 W, patients across a spectrum of diastolic dysfunction (11 age-matched controls, 9 with diabetes mellitus, 14 with clinical HFpEF, and 9 with amyloid cardiomyopathy) had a gradient of deficit in the PCr/ATP, an increase in the detected lung water again increasing across the groups, and (as expected) changes in atrial volumes that are likely associated with increased filling pressures.

**Fig. 7:**
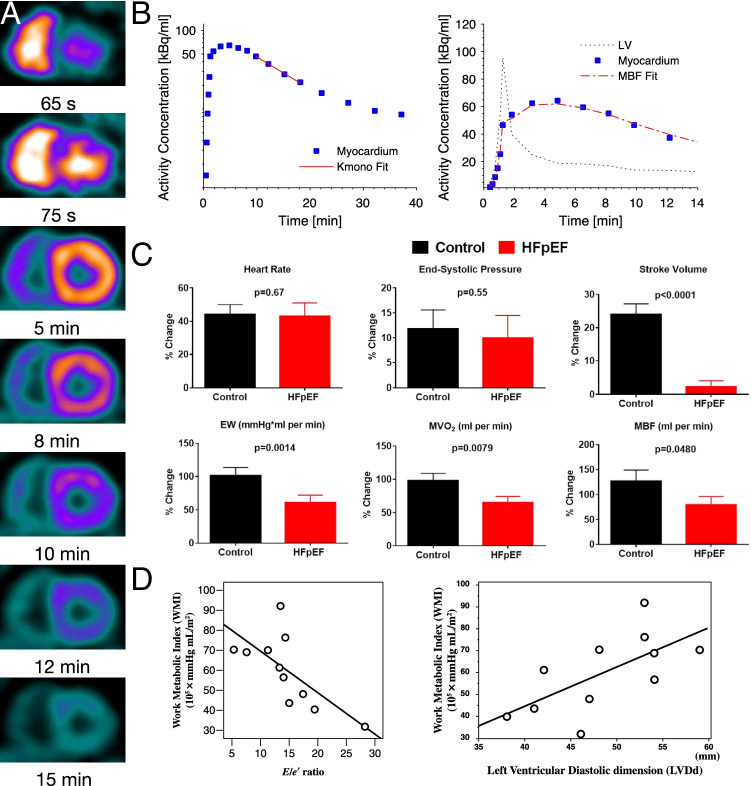
Myocardial oxidative metabolism inferred from the kinetics of the PET tracer ^11^C-acetate, which is visible in the RV and LV before being taken up into the myocardium and later excreted as it decays (**A**). By fitting these data to an appropriate mathematical model (**B**), it is possible to estimate myocardial mechanical work (denoted external work, EW) and infer MVO$_2$ and myocardial blood flow (MBF). In a trial of $n=19$ HFpEF patients undergoing dobutamine exercise-mimetic stress, it was found that the ability of the heart to augment these values was reduced in HFpEF. This is consistent with an earlier indication in $n=11$ HFpEF patients at rest that myocardial metabolic capacity is decreased as E/E’ increases (**D**) and as the left ventricular end-diastolic dimension decreases (LV EDV mass index is a diagnostic criterion of HFpEF), which highlights a metabolic impairment in the disease (panels **A**–**C** reproduced from [149]; **D** from [150] with permission)

These factors paint a strong picture of a maladaptive cardiac response to exercise that is associated with a potentially causative metabolic shift: as well as reporting reduced PCr/ATP ratios and increased exercise lung water, previous work has aimed to characterise cardiac metabolism in HFpEF with radiolabelled ^11^C-acetate as a PET tracer [150, 151]. Injected free acetate is rapidly taken up by the heart and then mitochondrially converted to acetyl coenzyme A and metabolised to carbon dioxide through the TCA cycle through oxidative phosphorylation; and thus, ^11^C-acetate PET imaging provides a reproducible proxy measurement of cardiac oxidative metabolism (i.e. MVO$_2$) at centres that have the cyclotron required to synthesise this tracer with a 20-min half-life. In a study of $n=11$ HFpEF patients and $n=10$ controls, decreased cardiac update of ^11^C-acetate was inversely correlated with E/e’ in patients only [150]. In a more recent study [149] that additionally included dobutamine-induced exercise mimetic stress with prospectively enrolled HFpEF patients ($n=19$) and matched controls ($n=19$) that evaluated myocardial blood flow (MBF) and mechanical cardiac work in addition to MVO$_2$, it was found that, at rest, compared with controls, patients with HFpEF had higher cardiac work, MVO$_2$, and MBF. During dobutamine stress, cardiac work, MVO$_2$, and MBF increased in both HFpEF and controls, but the magnitude of increases was significantly smaller in HFpEF. This is indicative, as before, of the impaired ‘exercise reserve’ of the HFpEF heart. In both groups, MBF increased with dobutamine stress in relation to cardiac work, but the magnitude of this increase was significantly reduced in HFpEF patients, while HFpEF patients with LV hypertrophy had a significant reduction in computed left ventricular mechanical efficiency compared to controls, summarised in Fig. 7. This implies a mismatch of both cardiac energetic demand and blood flow—pointing to some degree of coronary microvascular disease—and moreover an alteration in the metabolic efficiency of the heart. It is worth stressing that pulmonary hypertension alone has been indicated in driving the heart towards glycolysis and the role of the right-heart in HFpEF cannot be understated [152]—but, as ever, isolating cause, effect, and symptom remains challenging owing to the exquisitely integrated nature of cardiovascular physiology.

## A Future Role for Advanced Imaging in HFpEF?

An abnormal metabolism is clearly implicated in HFpEF and HFrEF alike, and the question of whether (or not) metabolic changes drive HFrEF is often described as a decades-old chicken-and-egg problem in the context of HFrEF [30]. The roles of the immune system, of titin, and of maladaptation to the resultant alterations in the mechanical stiffness of the HFpEF heart are also clear: once a patient has started down the path of diastolic dysfunction, increased filling pressures, and other comorbidities that define the HFA-PEFF diagnostic scoring system, the manifest condition is relatively easy to study. What is less clear, however, is exactly why the epidemiologically relevant risk factors for HFpEF are obesity and T2DM. HFpEF is not always the most straightforward of conditions to diagnose: relevant masquerading conditions to rule out via a number of distinct investigations include cardiac amyloidosis, sarcoidosis, hypertrophic cardiomyopathy, valvular heart disease, high-output heart failure, myocarditis, constrictive pericarditis, and the various toxin-mediated cardiomyopathies [153].

Advanced metabolic imaging, therefore, might fulfil two separate purposes. Firstly, in a clinical research or pre-clinical setting, the ability to determine uniquely the balance of substrates used by the myocardium would directly inform the research of the underlying shifts in cardiac metabolism and whether they precede, occur contemporaneously with, or follow alterations in myocardial stiffness (in HFrEF, myocardial metabolic changes precede the descent into heart failure [30, 154, 155]). Moreover, trials investigating pharmacologic regimens aimed at improving the functional outcomes of HFpEF patients, or prevent those at high risk from developing the disease, would benefit from the ability to have a direct readout of their metabolic effect. Many such therapies have been proposed and investigated for HF in general, with somewhat mixed effects: etomoxir and perhexiline (reported as targeting carnitine palmitoyl transferase, decreasing myocardial fatty acid metabolism, and favouring a reciprocal increase in glycolysis) have shown benefit in patients but may be limited by side effects; trimetazidine and L-propionylcarnitine, decreasing fatty-acid oxidation, increasing glucose oxidation; and increasing fatty acid transport across the mitochondrial membrane directly may have a survival and functional benefit [156]. Despite their success in pre-clinical trials, most of these molecules have not found widespread adaptation in patients. One potential reason for this is that “all-comers” trials based on those with a clinical deficit in functional scores such as the New York Heart Association functional class may not select patients with an underlying pathogenic mechanism immediately compatible with the metabolic modulation each drug provides. Being able to directly image the specific alterations involved in the disease is therefore highly desirable.

Secondly, beyond scientific inquiry into the molecular origins of HFpEF, there are several reasons to believe that multimodal metabolic imaging may have a patient-specific role in the diagnosis and management of the condition. Metabolic imaging—either in the form of advanced MRI or PET with appropriate tracers—has long been recognised of being valuable in cardiology where the metabolic demands of the organ are inherently linked to its function with disease-varied efficiencies [157–159]. Novel technological progress, in the form of either advanced PET reconstruction algorithms and improved multi-modal imaging, or hyperpolarised MR (which uses endogenous metabolites such as [1-^13^C]pyruvate together with low-temperature spin-physics to make their metabolism transiently visible to the scanner), heralds considerable promise for improving the ability of sites to routinely and rapidly quantify more aspects of cardiac function. For that reason, more than 42 clinical trials registered on clinicaltrials.gov (with 21 currently recruiting) aim to use advanced MR, multimodal cardiac MRI, and measures of metabolism directly to prognosticate HFpEF (for example, by quantifying its microvascular milieu through the use of first-pass perfusion gadolinium scanning at rest and under dobutamine stress, with radioactive H$_2$^15^O PET perfusion scans as a gold-standard perfusion readout [160]). Hyperpolarised MR, meanwhile, has already been shown to be of utility in investigating ischaemic heart disease [161], resolving both regional alterations in glucose utilisation caused by coronary artery disease, and in the diabetic myocardium, wherein insensitivity to glucose uptake and altered postprandial response was directly quantified [162]. The changes in energetic status within the HFpEF myocardium found via ${}^{31}$P spectroscopy are intriguing and point towards a disease on a spectrum of diastolic dysfunction potentially being both unified by an energetic deficit and ultimately prognosticated by it. Hyperpolarised pyruvate imaging can, in principle, achieve higher resolution imaging than similar PET scans within a minute [163] and may therefore be of utility in further characterising the HFpEF heart. This technique is currently in international clinical trials in a variety of indications, and offers the promise of a rapid and comprehensive assessment of myocardial metabolism which may be of benefit both in HFpEF and additionally for identifying the ischaemic region at risk in ischaemic cardiomyopathies. At present is a comparatively expensive and rare technique, but one with a wide number of potential applications both in cardiology and in other conditions, such as monitoring tumour response to therapy, and a similar cost base to PET. Should its value and utility be demonstrated conclusively in forthcoming trials, it is at least possible and at best probable that hyperpolarised MR could have clinical availability in the medium-term future.

The need for an advanced understanding of cardiac metabolism *in vivo* in human HFpEF patients is well illustrated by recent successful clinical trials. The surprising (yet positive) results of studies such as EMPA-REG which showed a significant benefit to cardiovascular mortality in T2DM patients with improved glycaemic control provided by empagliflozin, a sodium-glucose co-transporter 2 inhibitor [164], potentially by procuring a shift in cardiac metabolism [165], has led to a recent clinical trial aiming to replicate these findings in HFpEF patients independent of diabetes status. In the EMPEROR-Preserved trial of nearly 6000 patients with HFpEF and NYHA class II to IV functional impairment, empagliflozin reduced by around 21% the combined risk of cardiovascular death or hospitalisation for HFpEF patients, regardless of the presence or absence of diabetes [166]. This follows from a string of broadly speaking failures in wide HFpEF demographic groups: PEP-CHF (antihypertensive perindopril, [167]); CHARM-Preserved (antihypertensive candesartan, [168]), I-PRESERVE (antihypertensive irbesartan, [169]), TOPCAT (potassium-sparing diuretic spironolactone, [170]), and PARAGON-HF (angiotensin receptor-neprilysin inhibitor sacubitril/valsartan, [171]) all failed to show substantial benefit with hard patient end-points. The fact that a metabolic modulator, albeit one with a variety of as yet-to-be-elucidated cardiac effects, can significantly improve patient mortality in both HFpEF and HFrEF demographics is puzzling, and requires further scientific study. Even small beneficial changes in cardiac energetic utilisation within a beat-to-beat timeframe may translate into large differences in efficiency, known to be associated with outcomes [165]. The sister studies to EMPEROR-Preserved evaluating SGLT2 inhibition in HFrEF, the DAPA-HF and EMPEROR-Reduced trials, have similarly shown significant reductions in morbidity and mortality with SGLT2 inhibition (dapagliflozin and empagliflozin respectively), again independent of diabetic state [172–174]. This is strongly suggestive of an underlying metabolic mechanism, not least because the heart does not express SGLT2 and radiolabelled $^{14}$C autoradiographic studies indicate that it is not detectable directly in the heart [175]. Off-target effects involving either sodium homeostasis, or action on the sodium-proton exchanger, are not reported in isolated ventricular cardiomyocytes over a wide range of doses that may otherwise explain a direct cardiac mechanism of action [176]. The need to fully understand the metabolic changes that SGLT2 inhibitors can induce in patients with either HFrEF or HFpEF is therefore highlighted neatly: by altering systemic metabolism we can improve mortality, although we as yet do not know how.

The mechanisms underpinning the benefit of SGLT2 inhibitors (SGLT2i) in HFpEF remain elusive, though several compelling candidate pathways have been postulated. Restoration of myocardial energetics and normalisation of metabolism are a key candidate mechanism, linked to the observation that SGLT2is promotes mild ketosis, via an increase in production of the ketone body beta-hydroxybutyrate as reported in both invasive animal studies [177, 178] and patient populations [179, 180]. Ketones may offer a more efficient myocardial metabolic substrate, requiring fewer moles of oxygen per mole of ATP produced [181], thus counteracting metabolic inflexibility arising from an over-reliance on non-esterified fatty acids in HFpEF [182, 183]. Alternatively, ketones might abrogate pro-hypertrophic signalling pathways and prevent MAP kinase activation resulting in changes in left ventricular mass [184, 185]. Importantly, multiple facets of these mechanisms are amenable to non-invasive assessment, using the arsenal of imaging tools described in earlier sections. For example, this could include hyperpolarised ^13^C MRI and/or PET for cardiac substrate metabolism [186], ^31^P MRS for energetics, and ^1^H spectroscopy for myocardial lipid content. Ongoing clinical trials and investigations will aim to directly reproduce and test these findings in patient populations.

A second key candidate mechanism relates to modulation of the cardiac stroma, including resident innate immune cell and fibroblast function, which play a key role in maintaining normal cardiac function through the regulation of cardiac metabolism and the cardiac renin-angiotensin-aldosterone system, as well as the release of soluble paracrine factors with anti-fibrotic, pro-angiogenic, and anti-apoptotic effects [187, 188]. SGLT2is has been linked in other settings with amelioration of pro-inflammatory and pro-fibrotic signalling, and it is conceivable that these may have relevance to clinical benefit in HFpEF [189]. Again, using novel molecular imaging probes as described above in Sections “Imaging Inflammation” and “Cardiovascular MR in HFpEF”, it is now possible not only to assess key leukocyte populations within the myocardium, but also to measure myocardial fibrosis via cardiac extracellular volume. Additionally, the modulation of adipokine function (including a reduction in serum leptin) has been linked with altered epicardial fat deposition profiles [190, 191], the changes of which can be straightforwardly assessed using imaging.

Given the undeniable disease-modifying benefit that SGLT2i has been shown to have, elucidating their molecular mechanisms non-invasively with metabolic imaging methods in patients would provide a significant improvement in the understanding of HFpEF in general.

## Key Conclusions

HFpEF is a complex yet prevalent condition that is driven by an increase in the bio-mechanical stiffness of the heart, microscopically originating from changes in titin expression and alterations in the extracellular matrix and an increased collagen volume fraction of the myocardium. This leads to impaired relaxation, and hence maladaptive changes in the pressure/volume relationship of the heart. It is therefore often referred to as being defined by diastolic dysfunction.

Whilst sharing many common cardiovascular risk factors and co-morbidities with HFrEF, the HFpEF heart has itself been shown to be metabolically distinct. Advanced non-invasive imaging techniques are able to accurately quantify both the biomechanical phenotype of the HFpEF heart and its blunted haemodynamic response to exercise, and point towards energetic impairment. The recent emergence of SGLT2 inhibition as a disease-modifying therapy arguably provides further evidence that both myocardial and systemic metabolic effects should be investigated in order to further elucidate its mechanistic role in ameliorating this morbid and prevalent condition.

## Data Availability

Please see referenced primary manuscripts (if appropriate).

## Code Availability

Please see referenced primary manuscripts (if appropriate).
